# *In Vivo* Antitumor Effect against Murine Cells of CT26 Colon Cancer and EL4 Lymphoma by Autologous Whole Tumor Dead Cells

**DOI:** 10.1155/2021/6626851

**Published:** 2021-02-03

**Authors:** Carlos Barrera-Avalos, Ximena Díaz, Bastián Madrid, Sofía A. Michelson, Claudia Robles-Planells, Giselle Sánchez-Guerrero, Viviana Ahumada, Andrea Mella-Torres, Leonel E. Rojo, Mónica Imarai, Luis A. Milla, Elías Leiva-Salcedo, Paola Murgas, Ricardo Fernández, Alejandro Escobar, Claudio Acuña-Castillo

**Affiliations:** ^1^Departamento de Biología, Facultad de Química y Biología, Universidad de Santiago de Chile, USACH, Alameda, 3363 Santiago, Chile; ^2^Centro de Biotecnología Acuícola, Universidad de Santiago de Chile, USACH, Alameda, 3363 Santiago, Chile; ^3^Centro de Investigaciones Biomédicas y Aplicadas, Escuela de Medicina, Facultad de Ciencias Médicas, Universidad de Santiago de Chile, Chile; ^4^Center for Integrative Biology, Faculty of Sciences, Universidad Mayor, Santiago, Chile; ^5^Departamento de Salud, Universidad de Los Lagos, Osorno, Chile; ^6^Laboratorio Biología Celular y Molecular, Instituto de Investigación en Ciencias Odontológicas, Facultad de Odontología, Universidad de Chile, Santiago, Chile

## Abstract

Active immunotherapy against cancer is based on immune system stimulation, triggering efficient and long-lasting antigen-specific immune responses. Immunization strategies using whole dead cells from tumor tissue, containing specific antigens inside, have become a promising approach, providing efficient lymphocyte activation through dendritic cells (DCs). In this work, we generate whole dead tumor cells from CT26, E.G7, and EL4 live tumor cells as antigen sources, which termed immunogenic cell bodies (ICBs), generated by a simple and cost-efficient starvation-protocol, in order to determine whether are capable of inducing a transversal anticancer response regardless of the tumor type, in a similar way to what we describe previously with B16 melanoma. We evaluated the anticancer effects of immunization with doses of ICBs in syngeneic murine tumor models. Our results showed that mice's immunization with ICBs-E.G7 and ICBs-CT26 generate 18% and 25% of tumor-free animals, respectively. On the other hand, all carrying tumor-animals and immunized with ICBs, including ICBs-EL4, showed a significant delay in their growth compared to not immunized animals. These effects relate to DCs maturation, cytokine production, increase in CD4+T-bet+ and CD4+ROR-*γ*t+ population, and decrease of T regulatory lymphocytes in the spleen. Altogether, our data suggest that whole dead tumor cell-based cancer immunotherapy generated by a simple starvation protocol is a promising way to develop complementary, innovative, and affordable antitumor therapies in a broad spectrum of tumors.

## 1. Introduction

Active immunotherapy against cancer aims to trigger efficient and long-lasting, antigen-specific immune responses. Currently, different strategies have been developed in order to activate immune response against cancer, including the adoptive transference of autologous dendritic cells charged *ex vivo* with extract or whole tumor autologous or allogeneic cells [1, 2]. Whole tumor antigen offers an advantage as it allows DCs to process and present numerous tumor antigens to stimulate a strong T cell response to ameliorate tumor development. In theory, whole tumor cells could be used as a straightforward approach to vaccination and can be administered directly without preloading dendritic cells, generating simultaneous cytotoxic T lymphocytes (CTLs) and CD4^+^ T cell activation [3]. Therefore, an ideal vaccination method should simultaneously kill and enhance the immunogenicity of tumor cells to initiate an effective immune response. Cell death is a process that varies according to the cytotoxic stimulus; this influences immunogenic/anticancer potential of the cellular compounds generated during cell death. This has led researchers to coin the term immunogenic cell death (ICD) to refer to the type of death that best induces immunogenic responses [4, 5]. Immunogenic cell death depends largely on the death-initiating stimulus that could cause the exposure of immunogenic factors on the cell surface or the release of immunogenic signals into the extracellular space [6]. This can result from multiple cytotoxic stimuli and protocols, some of which have shown efficacy in clinical and preclinical experiments. A variety of chemotherapeutic drugs, such as anthracyclines and oxaliplatin [7], and also lethal doses of ultraviolet (UV) rays and gamma irradiation, to generating apoptotic tumor cells [8] can induce immunogenic tumor cells death.

Vaccines containing immunogenic whole dead tumor cells have been described as efficient strategies to generate antigen sources capable of increase DCs and T cell function. This was supported by Scheffer and coworkers [9], demonstrating that a vaccine based on dead irradiated CT26 cells induces *in vivo* protective immune response against tumors after a challenge with live CT26 tumor cells. Protective effects were also observed in animals vaccinated with apoptotic B16 [10] and colorectal cancer cells (CRC) killed by an oncolytic adenovirus [11]. Another example of this approach is the molecule Rose Bengal, which also induces immunogenic dead cells with antitumor effects against colorectal cancer cells [12]. Another method used to generate whole tumor cells is through autophagy induction, such as complete nutrient-starvation on culture cells [13]. This type of method was previously published by our group using B16 melanoma cells, the most aggressive skin cancer, using a simple and cost-efficient method [14]. Its subsequent administration in syngeneic mice improves antigen cross-presentation and induces *in vivo* protection against melanoma. In summary, literature consistently shows that whole cell-based vaccines generated through immunogenic protocols can induce efficient *in vivo* antitumoral responses.

In this work, we generated immunogenic whole dead tumor cells as antigens sources termed immunogenic cell bodies (ICBs), using a simple and cost-efficient starvation method described previously by us [14], compared to the others previously described, which can be reproduced in a wide spectrum of research laboratories [15, 16]. These ICBs were generated from different tumor cell lines, lymphoma EL4/E.G7 and colon cancer CT26. This was done in order to determine whether our ICBs are capable of inducing a broad-spectrum anticancer response, regardless of the tumor type, in a similar way to what we describe with B16 melanoma [13]. We aimed to expand the current therapeutic possibilities of whole cell-based immunotherapy against cancer through the use of starvation-induced dead cells.

## 2. Material and Methods

### 2.1. Animals

Male and female 6-to-12-week-old mice of the C57BL/6 (B6) or BALB/cJ strains were obtained from the University of Santiago of Chile research facility. Animals were maintained on ad libitum diet with cycles of 12 hours of light and 12 hours of darkness. Animal protocols were reviewed and approved by the Institutional Ethics Committee of the University of Santiago De Chile (Approval letter No. 598). All procedures were conducted in accordance with the guidelines for avoiding pain, distress, and discomfort in experimental animals.

### 2.2. Cells Cultures and Chemicals

CT26, EL4, and E.G7 (EL4-OVA) cell lines were maintained in cell culture conditions at 37°C in a humidified atmosphere under 5% CO_2_. Syngeneic bone marrow-derived dendritic cells (BM-DCs), hereafter called DCs, were generated from C57BL/6 mouse or BALB/c cells. Briefly, animals were sacrificed by cervical dislocation, and the femur and tibia were removed under sterile conditions. The proximal and distal ends of these bones were removed, and the bone was perfused with RPMI-1640 medium (Gibco ™). The cells obtained were centrifuged at 300 g for 10 minutes. The pellet was resuspended in ACK erythrocyte lysis solution (0.15 M NH_4_Cl, 10 mM KHCO, 0.1 mM EDTA) for 5 minutes with gentle agitation at room temperature, followed by centrifugation at 300 g for 7 minutes. The cell pellet was resuspended and seeded in a 24-well plate (1.0∗10^6^ cells per well) with RPMI-1640 medium supplemented with 10% inactivated fetal bovine serum (SFB; Gibco™), penicillin (100 U/mL)/streptomycin (100 *μ*g/mL), supplemented with 1 mM pyruvate (Gibco™), 1 mM L-glutamine (Gibco™), 1% amino acids no essential (Gibco™), 10 ng/mL of GM-CSF (Invitrogen™), and 10 ng/mL of IL-4 (Gibco™). After two days, 75% of the culture medium was renewed with both cytokines. Cells were used on the seventh day for each experimental assay. CD4 and CD25 antibodies were obtained from Santa Cruz Biotechnology (RM-4 and PC61 clones, respectively). CD8*α* (clone: 53-6.7), ROR-*γ*t (clone: Q31-378), T-bet (clone: 4B10), CD40 (clone: HM40-3), CD86 (clone: GL-1), MHC-I (clone: AF6-88.5.5.3), MHC-II (clone: M5/114.15.2), and CD11c (clone: N418) antibodies were obtained from BD Biosciences Pharmingen (San Diego, CA). T regulatory cell detection kit was obtained from eBioscience (San Diego, CA). The anti-mouse IL-12 p40 and anti-mouse TNF-*α* were purchased from Biolegend (clones: C15.6 and MP6-XT22, respectively). LPS (E. coli 026:B6) and peptide-OVA_257-264_ (SIINFEKL, S7951) were obtained from Sigma Aldrich. CellTracker™ red (CMTPX) was purchased from Invitrogen™.

### 2.3. Preparation of Immunogenic Cell Bodies (ICBs)

To obtain specific whole dead tumor cells, hereafter called ICBs, CT26, EL4, and E.G7 tumor cell lines were subjected to starvation as previously described [13]. Briefly, adherent cells were seeded and cultured up to 70% confluence or 0.5 × 10^6^ cell/ml, washed with phosphate buffer saline (PBS) and nutrient-deprived by switching from culture media to PBS containing 2.5 *μ*g/mL fungizone and 10 *μ*g/mL gentamycin, and incubating one week at 37°C in a humidified atmosphere under 5% CO_2_. At day seven, all cells were recollected and centrifuged at 400 g and washed, and the ICBs were used immediately for each experimental procedure. Cells were evaluated after seven days using cell cytometry analysis using propidium iodide and observed 100% of nonviable population. For the ICBs characterization, CT26 and EL4 cells were previously labeled with CellTracker™ red (CMTPX), respectively, and ICBs were generated as described previously. We used the Dead Cell Apoptosis Kit with Annexin V Alexa Fluor™ 488 and Propidium Iodide (PI) (Invitrogen™) to evaluate cell viability/apoptosis and confirm ICBs cell death, following the manufacturer's instructions. ICBs size and morphology were determined by confocal microscopy (Zeiss LSM 800 confocal microscope, Carl Zeiss, Inc). Images were analyzed using the Zeiss LSM 2.5 Blue software. The ICBs size was determined using the area of a circle formula (*A* = *π*∗*r*2).

### 2.4. Mouse Immunization and Tumor Induction

Animals either C57BL/6 or BALB/c were immunized subcutaneously (s.c.) with doses of ICBs generated from 2 × 10^5^ CT26, E.G7, or EL4 cells in the left flank without coadjuvants, once every seven days for three weeks. For negative immunization control, animals were immunized with the vehicle, which corresponds to PBS. One week after the last ICBs injection, animals were challenged (s.c. tumor induction), BALB/c with 2 × 10^5^ CT26 live cells and C57BL/6 with 5 × 10^5^ EL4 or E.G7 live cells. Tumor sizes were monitored daily with a caliper. When tumor reached a volume of 261 mm^3^, mice were sacrificed, and the spleen was removed. Tumor sizes were calculated using the hemisphere formula [*π*∗*r*^3^]∗2/3 where *r* is the tumor radius.

### 2.5. T Cell Population Analysis

At the end of the treatment (261 mm^3^ tumor size), animals were euthanized by cervical dislocation; the spleens were removed under sterile conditions and disaggregated in a metallic mesh (100 *μ*m). Splenocytes were obtained free of erythrocytes by treatment with ACK lysis buffer (NH_4_Cl 155 mM, KHCO_3_ 10 mM, Na_2_EDTA 1 mM, pH 7.3) with gentle agitation for 5 min, then centrifuged at 400 g for 10 min, cells were washed, and supernatant was discarded. Splenocytes were suspended at 1 × 10^6^ cells/mL in 1 mL of cold IF buffer (PBS 1X, 2% FBS) and incubated at 4°C for 30 min. Cells were then stained with antibodies against the cell surface markers with anti-mouse CD4-FITC (eBioscience), anti-mouse CD8-PE (eBioscience), and anti-mouse CD25-PE (eBioscience). Then, cells were resuspended in a fixation/permeabilization solution (Fix/Perm; eBioscience) and incubated with anti-mouse Foxp3-PerCP (eBioscience) antibody for Treg population, anti-human/mouse ROR-*γ*t-PE (eBioscience) antibody for Th17 population, and anti-human/mouse T-bet, PerCPCy5.5 (eBioscience) antibody for Th1 population, all simultaneously to anti-CD4-FITC antibody labeling (eBioscience, USA). All samples were analyzed by flow cytometry using a BD Accuri C6 cytometer (BD Bioscience, San Jose, California, USA).

### 2.6. Phagocytosis, Activation and Cross-Presentation by BM-DCs

For phagocytosis assay, BM-DCs from BALB/c or C57BL/6 were incubated for 24 hours with 2 × 10^5^ ICBs-CT26 or ICBs-EL4, respectively, previously labeled with CellTracker™ red (CMTPX) following the manufacturer's recommendations. BM-DCs were incubated with unlabeled ICBs as negative control or latex beads as positive control (Invitrogen™). After incubation, BM-DCs were collected, and 7-AAD staining was used to exclude dead cells, and CMTPX fluorescence was detected in total viable CD11c+ positive population by flow cytometry. To determine BM-DCs maturation, we challenged BM-DC with ICBs in a 1 : 2 ratio (DCs/ICBs) for 24 hours, and 1 *μ*g/ml LPS was used as positive control. To evaluate BM-DCs maturation, we used anti-mouse CD40 APC, anti-mouse CD86 APC, anti-mouse MHC-I FITC, anti-mouse MHC-II FITC, and CD11c PE for DCs lineage markers, all obtained from eBioscience (San Diego, California, USA). CD40, CD86, MHC-I, and MHC-II surface markers in BM-DCs were measured by flow cytometry. BM-DCs maturation graphics are represented by normalized mean fluorescence intensity (MFI), calculated by dividing the MFI of treatments by MFI of untreated or alone DCs (negative control). To evaluate cross-presentation, BM-DCs were stimulated for 24 h with under culture conditions with ICBs-E.G7 in a 1 : 2 ratio (DCs/ICBs). As positive control, BM-DCs were pulsed with 5 *μ*M OVA_257-264_ derivative peptide (SIINFEKL, S7951, Sigma) incubated for 180 min at 37° C in a 5% CO_2_ atmosphere, and BM-DCs incubated with ICBs-EL4 was used as negative control. After this time, cells were labeled with anti-mouse-CD11c-PE and anti-mouse OVA_257–264_ bound to H-2Kb/MHC-I APC– (SIINFEKL/H-2Kb, clone eBio25-D1.16, eBioscience), and SIINFEKL/MHC-I complex was detected on the surface of the BM-DCs by flow cytometry as cross-presentation parameter. For intracellular cytokine production, IL-12 and TNF-*α* analyses were performed. Syngeneic BM-DCs were generated as described above. BM-DCs were treated with Brefeldin A (Stem cell, Canada Inc., CA) at a final concentration of 10 *μ*g/mL to stop the vesicular transit. For BM-DCs treatment, 1 : 2 DCs/ICBs ratio was used. Untreated BM-DCs were used as negative control, and LPS was used as positive control. After 24 hours of BM-DCs stimulation, cells were collected and labeled with anti-mouse CD11c PE antibody at 4°C in darkness. Cells were then permeabilized and fixed with fixation/permeabilization solution (Fix/Perm; eBioscience) and labeled with anti-mouse IL-12 p40 and TNF-*α* APC antibodies. Finally, BM-DCs were resuspended in FACS buffer. All samples were analyzed by flow cytometry using a BD Accuri C6 cytometer (BD Bioscience, San Jose, California, USA).

### 2.7. Statistics

Tumor appearance was evaluated by the Kaplan-Meier method. Tumor growth was analyzed by a multiple *t*-test, followed by the Holm-Sidak method. A nonparametric Mann–Whitney test compared DCs maturation markers, cytokines production, and cross-presentation among different groups. Tumor growth was measured in all groups of animals when tumor size in the control group reached its maximum. The tumor size was evaluated with the two-tailed Fisher exact test and using contingency tables. The percentage differences between CD8+, CD4+, Treg, CD4+T-bet+, and CD4+ROR-*γ*t+ lymphocytes were analyzed with a Kruskal-Wallis ANOVA. All the analyses were performed using the GraphPad Prism 8.01 software. Results are presented as mean ± SEM, and statistical differences were considered significant at *p* < 0.05.

## 3. Results

### 3.1. ICBs-EL4 and ICBs-E.G7 Immunization Generates an Antitumor Effect against Lymphoma Model

**Figure 1:**
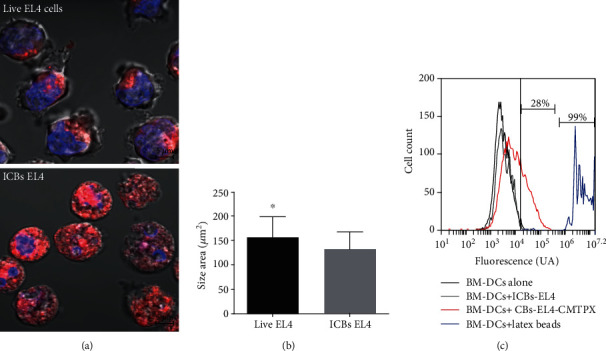
ICBs-EL4 characterization. (a) Size and morphology of live EL4 cells and ICBs-EL4 evaluated by confocal microscopy using CellTraker™ CMTPX Red and Hoechst 33342 shown at the top and bottom panels, respectively. (b) EL4 live cells and ICBs-EL4 area were measured, calculated, and represented as average ± standard error. (c) The ability of DCs to phagocyte ICBs-EL4 was evaluated using syngeneic BM-DCs cells (DCs) generated as described in methods. DCs were challenged with 1 × 10^5^ ICBs-EL4 stained with CellTraker™ CMTPX Red. Representative histograms show the CD11c+ population that acquired CMTPX fluorescence from the ICBs-EL4 (red peak), negative control is showed with DCs alone (black peak) and DCs-ICBs-EL4 without staining (grey peak). Latex beads were used as positive control (peak blue). 7-AAD+ cells were excluded to avoid detection of nonspecific ICBs bound to DCs. The asterisks (^∗^) represent statistically significant effects (*p* < 0.05). *n* = 4 − 12.

We have reported that dead cells generated by simple serum-starvation from B16 melanoma, termed here ICBs, are capable of inducing an antitumor response against melanoma *in vivo* [13] (Barrera-Avalos, submitted 2020). In order to determine whether this antitumor effect of our ICBs generated are reproducible in other types of cancer cells, we evaluated the effect on lymphoma model EL4 and EG-7 (EL4 expressing OVA). We used our starvation protocol previously reported, to determine the possible antitumor effect *in vivo* of these ICBs against the EG.7 lymphoma model. First, we physically characterized the ICBs-EL4, regarding their aspect and size with respect to live EL4 cells. As shown in Figure 1(a), bottom panel spherical cell-like structures (ICBs) were observed after 7 days of starvation. These structures showed irregular membranes, collapsed nuclei, and cell breakdown as compared with live EL4 cells (Figure 1(a), top panel).  Figure 1(b) showed that these ICBs are significantly smaller than those of live EL4 cells and are phagocyted by syngeneic BM-DCs. This was concluded because the CMTPX fluorescence from ICBs-EL4 was detected in live CD11c+ population (red peak) in a 28% approximately, in comparison with ICBs-EL4 without CMTPX (grey peak) (Figure 1(c)). The phagocytosis of antigenic tumor sources is a crucial stage to inducing an immune response *in vivo.* These structures yielded, along with the released extracellular vesicles, tumor antigen sources for our anticancer vaccine.

**Figure 2:**
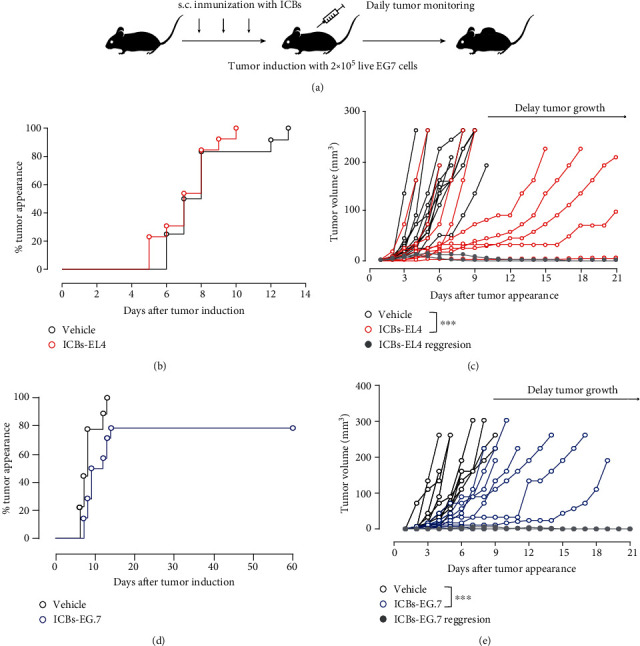
ICBs-EL4 induce an antitumoral effect against E.G7 lymphoma model. (a) Schematic representation of the immunization protocol. C57BL/6 mice were immunized with either 2 × 10^5^ ICBs-E.G7 or ICBs-EL4 every seven days for three weeks. One week after the final immunization, the animals were challenged subcutaneously (s.c.) with 2 × 10^5^ live E.G7 cells. Tumor was evaluated daily, and its appearance was graphed with Kaplan-Meier with % tumor appearance curves in ICBs-EL4 immunized animals (red circles), ICBs-E.G7 ((d) blue circles), and control animals (black circles, vehicle-PBS). E.G7 tumor growth in control and immunized animals with ICBs-EL4 and ICBs-E.G7 that develop tumor (c, e), respectively. The grey line represents animals with complete tumor regression. The asterisks (^∗∗∗^) represent statistically significant effects (*p* < 0.001). *n* = 9 − 13.

To determine the effect of ICBs-EL4 *in vivo* against the EG.7 lymphoma model, C57BL/6 animals were immunized with ICBs-EL4 and challenged with live EG.7 (EL4-OVA) as shown in Figure 2(a). ICBs-EL4 immunized animals did not protect and all animals develop detectable tumors between 10 and 13 days in the control group and ICBs-EL4 immunized group (red circles), respectively (Figure 2(b)). However, interestingly, the treatments with ICBs-EL4 induced 18% of complete tumor regression (grey line), and tumor growth rate was delayed in 67% of the animals, compared to the control group (Figure 2(c)). We also evaluated the effects of immunization with ICBs-E.G7 and challenged with live EG.7 tumor cells. As shown in Figure 2(d), nonimmunized animals generated tumors at days 6 to 14 after challenge and immunized yielded 20% tumor-free animals.  Figure 2(e) shows that ICBs-EG.7 immunization induced 18% of complete tumor regression (grey line) and significantly delayed tumor growth in 80% of the animals (Figure 2(e)). These data suggest that ICBs-E.G7 displays antitumor effects by specific OVA antigen.

### 3.2. ICBs-EL4 Induce Maturation and a Splenic Increase of CD4+Ror*γ*t+ Lymphocyte Populations

**Figure 3:**
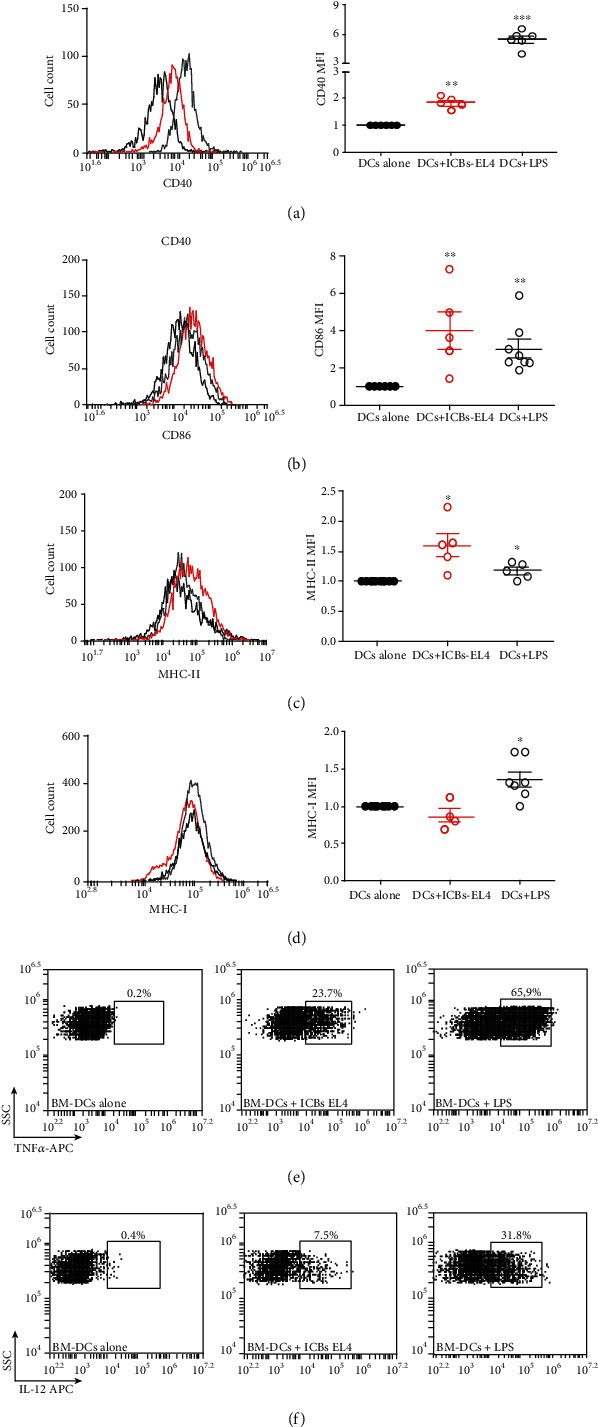
ICBs-EL4 lead maturation of syngeneic BM-DCs. The effect of ICBs-EL4 on levels of CD40 (a), CD86 (b), MHC-I (c), and MHC-II (d) markers and TNF*α* (e) and IL-12 (f) cytokine in syngeneic BM-DCs after 24 hrs of stimulation. BM-DCs challenged with EL4-ICBs correspond to red circles, untreated DCs correspond to black circles, and LPS treated correspond to open circles. Graphics show normalized median fluorescence intensity (MFI); this was calculated as a ratio between the MFI of the DCs treated with ICBs-EL4 and the MFI of the untreated DCs or DCs alone. TNF*α* (e) and IL-12 (f) cytokine productions are shown in representative histograms for untreated DCs (left panels), BM-DCs challenged with ICBs-EL4 (middle panels) and with LPS (right panel). Representative histograms are shown in all cases. Grey lines represent the LPS, black line corresponds to untreated DCs as negative control, and red line corresponds to the treatment with ICBs-EL4. Data are expressed as mean ± SEM. The asterisks (^∗^) and (^∗∗^) represent statistically significant effects (*p* < 0.05) and (*p* < 0, 01) respectively. *n* = 5 − 8.

**Figure 4:**
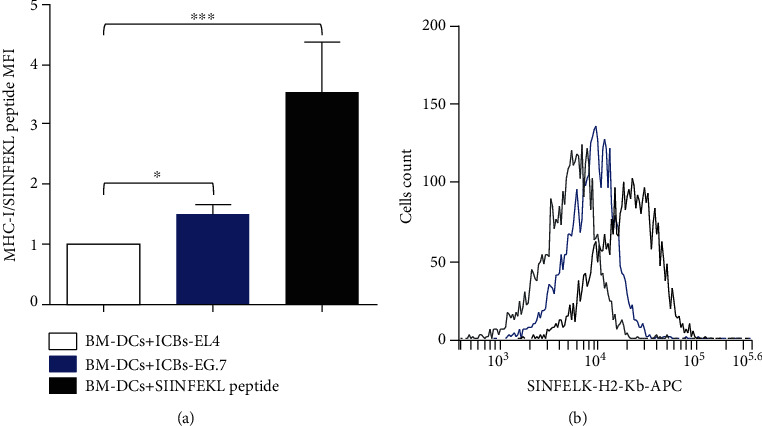
ICBs-EG.7 induce cross-presentation. Cross-presentation was evaluated by detection of the SIINFEKL (OVA_257-264_)/MHC-I complex on the surface of BM-DCs. (a) Graph includes 5 independent experiments, and data are shown as normalized mean fluorescence intensity (MFI), calculated as a ratio of the MFI of the BM-DCs stimulated with ICBs-E.G7 with the MFI of DCs-ICBs-EL4 (control). (b) In the representative histogram, DCs challenged with ICBs- EL4 (grey line), DCs challenged with E.G7- ICBs are shown in blue, and DCs pulsed with SIINFEKL peptide were used as positive control (black line). The data are expressed as mean ± SEM. The asterisks (^∗∗^) and (∗∗∗) represent statistically significant effects (*p* < 0.01), (*p* < 0,001), respectively.

In order to provide the first lights of a possible mechanistic explanation for the antitumor effect of these ICBs, we aimed to determine whether ICBs from EL4 cells would also generate maturation of syngeneic BM-DCs *ex vivo*. As shown in Figure 3, ICBs-EL4 significantly increased MHCII, CD86, and CD40 molecule expressions (Figures 3(b)–3(d), respectively), but not MHCI (Figure 3(a)) in BM-DCs. In all cases, except for CD40 marker, expression levels were similar to those of LPS-treated cells as a positive control. Also, ICBs-EL4 induced functional activity of BM-DCs evidenced by the increase of intracellular TNF-*α* and IL-12 cytokine production after ICBs-EL4 challenge (Figures 3(e) and 3(f), respectively). Syngeneic BM-DCs, challenged with ICBs-E.G7, increase significantly the SIIINFEKL (OVA_257-264_), or MHC-I complex is on the surface of BM-DCs compared to the negative control of ICBs-EL4 (without OVA), indicating that our ICBs generate antigen cross-presentation *in vitro* (Figure 4). This proves that DCs internalize, process, and present in MHC-I context, the OVA antigen from, suggesting that this maturation and antigen presentation as a putative mechanism involved in the antitumor described effect.

**Figure 5:**
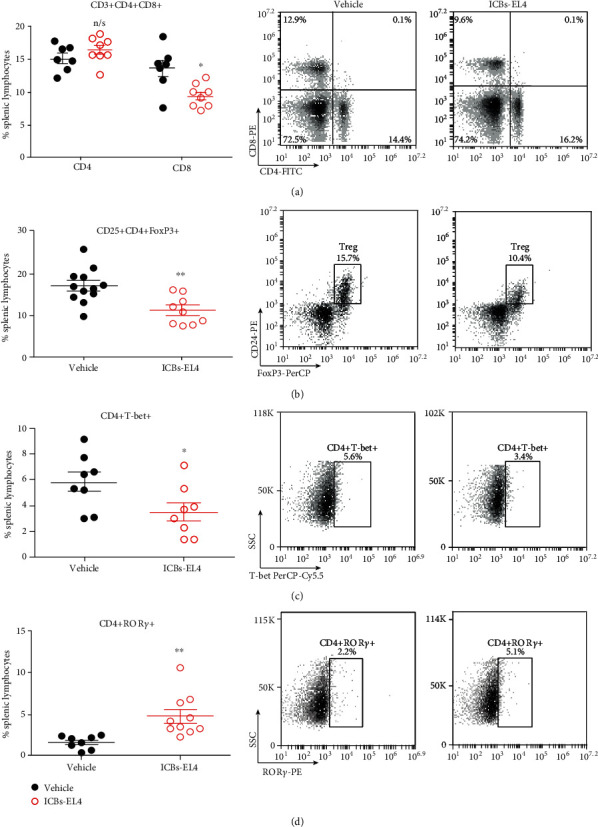
Treatment with ICBs-EL4 induces a reduction in splenic CD4+CD25+FOXP3+ Treg and increase CD4+T-bet+ populations. Spleens from 8 to 15 animals immunized with ICBs-EL4 were obtained at the end of treatment, and splenic T lymphocytes were evaluated as described in methods. The results are presented as graph (left panel) and dot plots (right panels). Vehicle (black circles) and ICBs-EL4 (red circles). 3 × 104 total events were acquired for analyses of (a) CD4+ and CD8+, (b) CD4+CD25+FOXP3+, (c) CD4+ T-bet, and (d) CD4+ROR-*γ*t+ lymphocytes. Data are expressed as mean ± SEM. The asterisks (^∗^) and (^∗∗^) represent statistically significant effects (*p* < 0.05), (*p* < 0, 01), respectively. n/s means not significant effect.

The possible mechanism involved in our results could include antitumoral response by T cells activation and polarization, in a similar way to that described with other types of tumor vaccines [3]. At the end of treatments, CD8+ and CD4+ conventional populations were unchanged, but we observed an increase of CD4+ROR-*γ*t+ (markers compatible with Th17 population) and decrease of CD4+CD25+FoxP3 (Treg) subpopulations in the spleen, as shown in Figure 5. Altogether, those results suggest that immunization induced the activation and balance of cellular immune responses.

### 3.3. ICBs-CT26 Immunization Induces Tumoral Protection against CT26 Tumor Model

**Figure 6:**
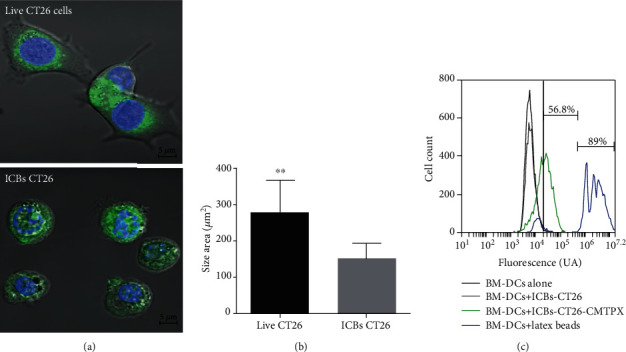
ICBs-CT26 characterization. (a) Size and morphology of live CT26 cells and ICBs-CT26 were evaluated by confocal microscopy using CellTraker™ CMFDA Green and Hoechst 33342 shown at the top and bottom panels, respectively. (b) CT26 live cells and ICBs-CT26 area was measured, calculated, and expressed as average ± standard error. (c) The ability of the DCs to phagocytose ICBs-CT26 was evaluated using syngeneic BM-DCs cells (DCs) generated as described in methods. DCs were challenged with 1 × 10^5^ ICBs-CT26 stained with CellTraker™ CMTPX (pseudocolor green). Representative histograms show the CD11c+ population that acquired the CMTPX from the ICBs-CT26 (green peak). Negative control is showed with DCs alone (black peak) and DCs-ICBs-EL4 without staining (grey peak). Latex beads were used as positive control (blue peak). 7-AAD+ cells were excluded to avoid detection of ICBs-CT26 unspecific bound to DCs. The asterisks (^∗∗^) represent statistically significant effects (*p* < 0.01). *n* = 4 − 12.

Our next aim was to evaluate whether ICBs are capable to induce antitumoral response on a BALB/c colon carcinoma tumor model CT26 cell line. We obtained ICBs-CT26 using the starvation protocol and demonstrated that ICBs-CT26 showed spherical cell-like structures with irregular membranes and collapsed nuclei (Figure 6(a), bottom panel) compared with live CT26 cells (Figure 6(a), top panel). We also used cell cytometry analysis using propidium iodide, observing 100% of nonviable population. The starvation method also induced significantly smaller structures compared with those from live CT26 cells (Figure 6(b)). ICBs-CT26 are phagocyted by syngeneic BM-DCs because his CMTPX fluorescence was detected in live CD11c+ population (green peak) in a 57% approximately, in comparison with ICBs-CT26 without CMTPX (grey peak) (Figure 6(c)). These results suggest that both ICBs-CT26 and ICBs-EL4 are similar in terms of morphology and phagocytosis induction by BM-DCs.

**Figure 7:**
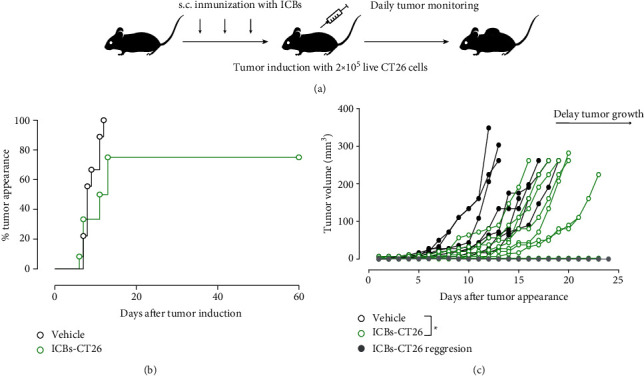
ICBs-CT26 protect mice against CT26 tumor. (a) Schematic representation of the immunization protocol. BALB/c mice were immunized with 2 × 10^5^ ICBs-CT26 every seven days for three weeks. One week after the final immunization, the animals were challenged with 2 × 10^5^ live CT26 cells. (b) Tumor was evaluated daily, and its appearance was graphed with Kaplan-Meier with % tumor appearance curves in ICBs-CT26 immunized animals (green circles) and control animals (black circles; vehicle is PBS). (c) CT26-tumor growth of control and immunized animals with ICBs-CT26 that developed tumor. The grey line represents animals with complete tumor regression. Tumor growth was measured in all groups of animals when tumor size in the control group reached its maximum. The tumor size was evaluated with the two-tailed Fisher exact test and using contingency tables. The asterisks (^∗∗∗^) represent statistically significant effects (*p* < 0.001). *n* = 9 − 12.

After verifying that BM-DCs efficiently phagocytize our ICBs-CT26, we followed the immunization and tumor challenge protocol shown in Figure 7(a). Under our experimental conditions, all animals of the control group developed tumors on day 12 after. Interestingly, the group of animals immunized with syngeneic ICBs-CT26 showed 25% of tumor-free animals (green circles) (Figure 7(b)). All tumor-developing animals are shown in Figure 7(c), where 36% of the group immunized with ICBs-CT26 induced a significant delay compared to the non-immunized group (black circles, vehicle). Furthermore, 8% of the vaccinated animals showed a total regression of the tumor (grey line). These *in vivo* results indicate that our ICBs-CT26 induce tumor protection and growth delay in colon cancer, confirming that the protective effect is not restricted to one particular type of tumor.

**Figure 8:**
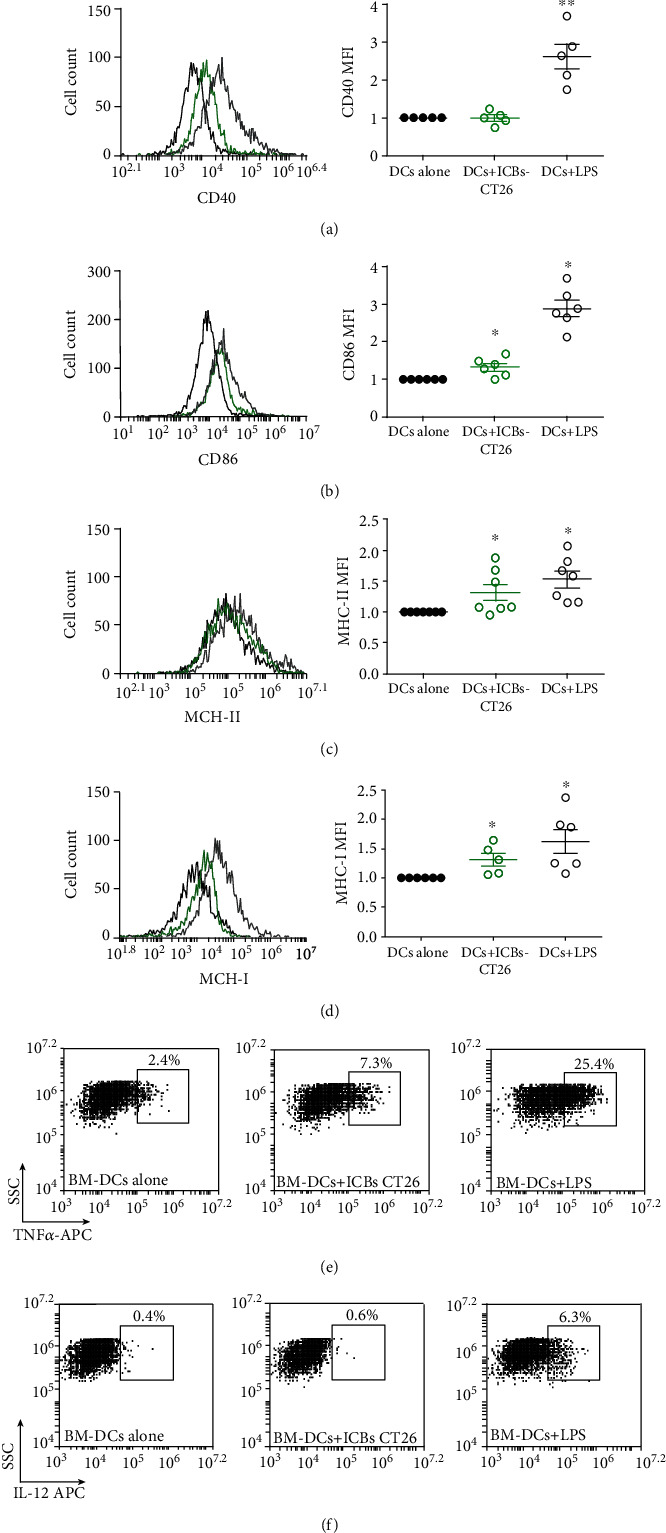
ICBs-CT26 lead maturation of syngeneic BM-DCs. The effect of ICBs-CT26 on levels of CD40 (a), CD86 (b), MHC-I (c), MHC-II (d) markers, and TNF*α* (e) and IL-12 (f) cytokine in syngeneic BM-DCs after 24 hrs of stimulation. BM-DCs challenged with CT26-ICBs correspond to green circles, untreated DCs correspond to black circles, and LPS treated correspond to open circles. Graphics show normalized median fluorescence intensity (MFI); this was calculated as a ratio between the MFI of the DCs treated with ICBs-CT26 and the MFI of the untreated DCs or DCs alone. TNF*α* (e) and IL-12 (f) cytokine productions are shown in representative histograms for untreated DCs (left panels), BM-DCs challenged with CT26-ICBs (middle panels), and with LPS (right panel). Representative histograms are shown in all cases. Grey lines represent to LPS, black line corresponds to untreated DCs as negative control, and green corresponds to the treatment with ICBs-CT26. Data corresponded to at least 5 independent experiments and were expressed as mean ± SEM. Asterisks (^∗^) represent statistically significant effects (*p* < 0.05).

**Figure 9:**
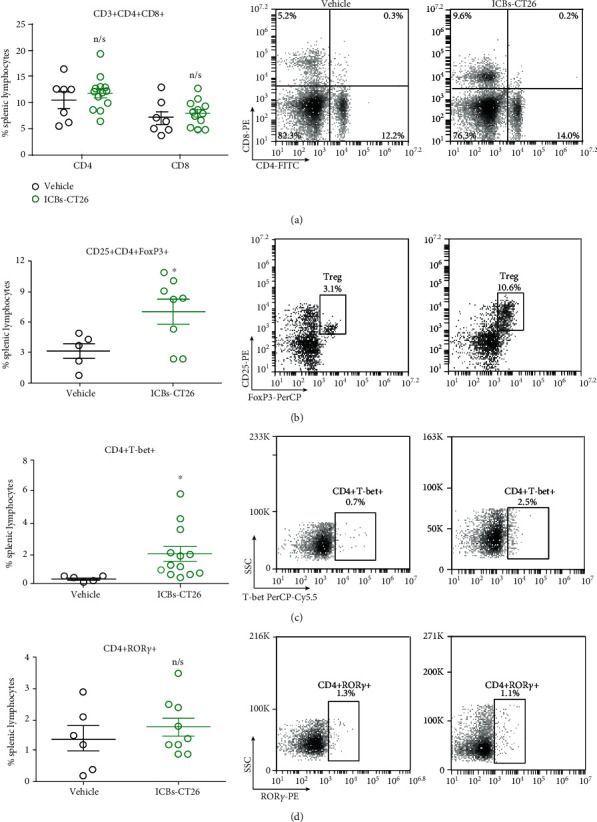
Treatment with ICBs-CT26 induces an increase in splenic CD4+CD25+FOXP3+ Treg and increase the CD4+T-bet+ populations. Spleens from 5 to 14 animals immunized with ICBs-CT26 were obtained at the end of treatment, and splenic T lymphocytes were evaluated as described in methods. The results are presented as graphs (left panel) and dot plots (right panels). Vehicle (black circles) and ICBs-CT26 (green circles). 3 × 10^4^ total events were acquired for analyses of (a) CD4+ and CD8+, (b) CD4+CD25+FOXP3+, (c) CD4+ T-bet, and (d) CD4+ROR-*γ*t+ lymphocytes. Data are expressed as mean ± SEM. The asterisk (^∗^) represents statistically significant effects (*p* < 0.05); n/s means not a significant effect.

Similar to the results obtained in the C57BL/6 mice lymphoma model, BM-DCs from BALB/c were activated and matured by syngeneic ICBs-CT26. As shown in Figure 8, ICBs-CT26 increased levels of MHCI and MHCII, similar to those obtained in LPS-treated cells (Figures 8(c) and 8(d), respectively). However, although the levels for CD86 and CD40 increased significantly, they were lower than those of LPS treatments. Similar to BM-DCs with ICBs-EL4, syngeneic ICBs-CT26 induced only an increase in TNF-*α* production (Figure 8(e)). Altogether, these results suggest that the *in vivo* antitumor mechanism might involve effective DCs activation and, therefore, trigger an increased immune response. In contrast with the lymphoma models, ICBs-CT26 treatment did not induce changes in classical CD8+ nor CD4+ populations, however did induce significant increase of CD4+CD25+FoXP3 and CD4+ Tbet+ subpopulation in the spleen, without changes in CD4+ROR-*γ*t+ subpopulations (Figure 9). All these results indicate that our starvation protocol for the generation of ICBs as antitumor vaccine works well in a different type of cancer cells.

## 4. Discussion

We determined that immunization with ICBs whole tumor cell vaccine generated by starving *in vitro* cultured tumor cell lines E.G7, EL4, and CT26 induce an 18-25% rejection and some delay in growth of some tumors that did grow. Previously, we reported that ICBs that express fusogenic protein of the infectious salmon anemia virus (ISAV) could activate DCs *in vitro* and induce antitumoral responses in a B16 melanoma model (Barrera-Avalos 2020). Our results showed a modest antitumor effect in all the tumor models evaluated, which correlated with efficient phagocytosis by DC, inducing increased CD86/40 costimulation molecules and releasing TNF-*α* and IL-12 cytokines. These events, along with others, are crucial for general *in vivo* antitumor responses [17]. These effects were also correlated with an induction of T cell population associated with cellular responses and a decrease in the regulatory population in treated animals compared to the nonimmunized mice. Our results agree with that obtained by the group of Briones, where they used a complex antitumor vaccine composed of DC fused with CD40L-transfected murine lymphoma tumor cells. They observed DCs maturation, tumor protection, and polarization of the CD4+ T cell population towards the Th17 population [18]. On the other hand, the reduction in immune suppressor cells, such as myeloid-derived suppressor cells and regulatory T cells, is associated with good clinical prognosis in patients with non-Hodgkin lymphoma [19]. Our study is also in agreement with that Hus et al., who described that a polarization towards Th17 lymphocytes and a decrease in regulatory T was crucial in patients who received vaccines for a mixture of antibody compounds and chemotherapeutic drugs [20]. Galan and collaborators indicated that the Th17 population is important in response against a non-Hodgkin lymphoma model [21]. Our results are comparable with other similar approaches. A similar B-cell lymphoma model used adoptive transfer of lymphoma antigen-loaded DCs vaccine, generating antitumor protection [22, 23]. Zappasodi and collaborators showed that a combinatorial treatment involving a vaccine generated by DCs loaded with apoptotic autologous tumor cells, induced by heat shock, *γ*-ray, and UV radiation, improvement in 33% of relapsed B-cell lymphoma patients (Zappasodi et al. 2010). This process is similar to those of chemotherapeutic agents that induce immunogenic cell death [24], for instance, the use of oxoplatinum to generate ICD in EG7 model (EL4 expressing OVA cells) and N-(2-Hydroxypropyl) methacrylamide, a doxorubicyn-conjugated copolymer, to induce a strong protection rate against tumors in the EL4 cell model [25]. Although an increase in the CD8+ T lymphocyte population is important for the antitumor immune responses, we believe that the decrease in CTL observed in the spleen is not necessarily associated with lower tumor-infiltrating lymphocytes (TILs). Despite this, we were able to determine that our ICBs-EG.7 induces cross-presentation of antigens, an event that is crucial in CD8+ activation and antitumor response [26]. It does not escape to our attention that there are previous reports showing that complete vaccines generated by DCs with whole-cell inductors or other compounds like antibodies, together with chemotherapeutic drugs that are capable of protecting against tumor growth. However, this work is the first report where a simple vaccine is used to protect against lymphoma.

As we stated before, whole-cell vaccines have been widely used and have shown good results in the CT26 cell line, but in other cell lines, only mild protective effects have been achieved ([27]); however, the combinatorial approach appears to be more including dead syngeneic CT26 cells with anthracycline [8]. These responses seem to depend on the death-induction mechanisms and other factors that may increase immune responses. For example, Je-Jung Lee's group using a vaccine generated by DCs loaded with whole dead tumor cells from MC-38 cell line, a colon carcinoma generated by gamma radiation, reported a significant delay in autologous tumor growth, depending on a polarization of CD4+ towards Th1 (T-bet+CD4+) and DCs maturation, in the same way as we reported in this investigation [28]. On the other hand, the administration of whole tumor cell vaccines composed of CT26 cells killed by incubation with anthracycline drugs generates tumor protection and phagocytosis by dendritic cells in a calreticulin-dependent manner [29]. In addition, other groups reported that their whole-cell vaccine generated by CT26 cells killed by freeze-thaw cycles in conjunction with baculovirus, induces tumor protection and delay, along with increased phagocytosis by DC [30]. All these reports are in agreement with what reported here using our ICBs-CT26. Although CD4+ and CD8+ cells did not have changes in spleen levels, we cannot rule out that their increase is probably due to intratumoral infiltration. Although in the present work, the treatment induced slight changes in the analyzed T cell populations; the time points used for the analysis could have masked possible changes on T cell populations induced by treatments. This was actually observed with the Treg population in ICBs-CT26 treatment, where an increase is made at poor prognosis. Thus, it is possible that at the time of analysis, the tumor might have overcome immunological restrictions imposed during the equilibrium phase, leading to the final growth.

The possibility to extrapolate our result to humans or clinical trials is far and could be oriented to pet treatments. However, prior works have documented the effectiveness of using whole dead tumor cells to promote and increase an *in vivo* antitumor effect, either through direct administration or preloaded on dendritic cells *ex vivo* [31]. For instance, Schmidt C's group examined 173 tests of clinical immunotherapies conducted by researchers using immunodominant synthetic antigens, allogeneic, and autologous whole tumor cells, without concomitant therapies in patients with prostate, breast, colorectal, melanoma, and lung cancer. This report noted that patients who were immunized with whole tumor cells showed effective clinical responses (8.1%) compared to patients immunized with synthetic peptides or proteins and viral or plasmid vectors encoding proteins (3.6%) [32], demonstrating the promising use of whole tumor cells as an antitumor vaccine. On the other hand, other vaccines are generated from the incubation of whole tumor cells with dendritic cells *in vivo*, which are adoptively transferred to an autologous patient. This type of vaccine has had good results in clinical trials. For example, the use of whole tumor lysate as cancer vaccine in a clinical trial of 43 stage IV and 7 stage III melanoma patients vaccinated with autologous DCs pulsed with an allogeneic cell lysate derived from three melanoma cell lines showed more than 60% of the stage IV patients had positive delayed-type hypersensitivity (DTH) reaction after vaccination, and the median survival of these patients was 33 months compared to 11 months for stage IV patients without DTH response. All stage III patients were DTH-positive after vaccination and remained tumor-free for a median follow-up period of 48 months [33]. The efficacy of vaccination with DCs loaded with whole tumor cells had similar clinical efficacy in other types of tumors [34]. These results were closely related to an increase in the immune response through CD4+ and CD8+ T. Also, Li and coworkers used macroautophagy triggered of HEK 293T cells expressing an antigenic model of chicken egg albumin (Ovalbumin or OVA) showed enhanced OVA to CD8+ T cells cross-presentation by dendritic cells [35]. However, many of the vaccines contemplate complex and expensive methodologies with specific equipment and components, making them less massive or difficult to access cancer patients.

Our simple and cost-effective method allows the production of whole dead tumor cell vaccine from any cancer cell line. This experimental approach significantly delays tumor growth in all the model systems studied, even with regression in some cases, suggesting that our ICBs may have a broad spectrum of antitumor action. This kind of strategy may complement other therapies such as the inhibition or blocking of immune system checkpoints (CTLA-4 and PD-1/PD1L axis) and may be used as an attractive combinatory strategy [36]. This is susceptible to experimental testing using clinical monoclonal antibodies like Ipilimumab and Nivolumab/Pembrolizumab, respectively, which have shown positive results in some forms of lymphomas [37] and are still being studied for CRC treatment [38]. This suggests that combined treatments could be the best clinical option. Meanwhile, the “immune responses” and protection could be considered modest even with 3 preimmunizations. We also propose that the use of agents capable of delaying tumor development such as our EL4-ICBs and CT26-ICBs, altogether with other canonical treatments, may help to widen the window for therapeutic options for cancer patients.

## Data Availability

Data is available on request.

## Nomenclature

DCs: Dendritic cells
BM-DCs: Bone marrow-dendritic cells
ICBs: Immunogenic cells bodies
EVs: Extracellular vesicles
ICD: Immunogenic cell death
